# Biogenetic ties and parent‐child relationships: The misplaced critique

**DOI:** 10.1111/bioe.12621

**Published:** 2019-08-06

**Authors:** Timothy F. Murphy

**Affiliations:** ^1^ Department of Medical Education University of Illinois College of Medicine at Chicago Chicago Illinois

**Keywords:** assisted reproduction, children, ethics, genetic relatedness, same‐sex couples

## Abstract

According to an almost axiomatic standard in bioethics, moral commitment should ground parents’ relationship with their children, rather than biogenetic relatedness. This standard has been used lately to express skepticism about extending existing assisted reproductive treatments (ARTs) to same‐sex couples and to research into novel fertility interventions for those couples, but this skepticism is misplaced on several grounds. As a matter of access and equity, same‐sex couples seem presumptively entitled to genetic relatedness to their children as far as possible both in regard to existing ARTs and to novel ARTs under investigation. For those worried about the effects of trying to secure biogenetic relatedness for same‐sex couples, it may be noted that same‐sex couples will only ever be a fraction of the parents implicated in propping up “biologism,” as the expectation of biogenetic relatedness it is sometimes called. The cultural force of biologism would survive almost intact even if no same‐sex couples were ever to have genetically related children. It is therefore hard to see why same‐sex couples should forfeit aspirations to biogenetic relationships with their children or enjoy less subsidy for ARTs than the subsidy given to different‐sex couples. As matter of moral consistency, the full implications of the biologism critique have yet to be evaluated relative to different‐sex couples.

## 1 INTRODUCTION

A nearly axiomatic account prevails in bioethics that parent‐child relationships should depend for their value on parents’ moral and psychological commitment to the children, not on biogenetics, by which I mean a genetic and/or gestational relationship. Thomas Murray has expressed the view this way: “Genetic parenthood is incidental to parent‐child mutuality.”1Murray, T. (2005) Three meanings of parenthood. In M. A. Rothstein, T. H. Murray, G. E. Kaebnick, & M. Anderlik Majumder (Eds.), *Genetic ties and the family: The impact of paternity testing on parents and children *(pp. 18–34)*.* Baltimore, MD: Johns Hopkins University Press. In fact, some commentators have even argued that certain harms to children have their origin in biogenetics, or “biologism” as it is sometimes called.2Roache, R. (2016). The value of being biologically related to one’s family. *Journal of Medical Ethics,* *42*(12), 755–756. This account is not universally shared, but it is often treated as self‐evidently true in certain evaluations of family relationships, fertility treatments, and research programs.3For a contrarian view, see Velleman, J. D. (2005). Family history. *Philosophical Papers,* *34*(3), 357–378. When applied to same‐sex couples’ interests in biogenetically related children, these evaluations typically unfold as follows: Commentators describe some prospect for giving each member of same‐sex couples an option for biogenetic ties to their children. Such options might involve, for example, synthetic gametes (with one male offering naturally produced sperm and his partner offering synthetic ova, or one female offering naturally produced ova and her partner other offering synthetic sperm) or shared maternity (in which one female partner offers the ova for conception and the other female partner gestates the child). The commentators then typically point out that parental interest and/or responsibilities should not depend on biogenetics. Various recommendations are then given: to counsel people against trying to secure genetically related children, to counsel them against having children at all, or to authorize the withdrawal of state support for clinical treatment and/or research that presupposes biogenetic relationships to children. These recommendations are, in sum, held out as ways to undercut the morally dubious interpretation of children as desirable because of their biogenetic relatedness to parents.

I will argue that this line of analysis amounts to a misplaced critique in its focus on same‐sex couples. If skepticism toward genetic relatedness is credible, if biogenetic relatedness should not matter in a parent’s relationship to a child, and if society should not act in ways that reinforce biologism, the deeply embedded social practices that sustain and support biologism in *different‐sex* couples ought to shoulder the brunt of the critique. At the very least, it remains morally undefended that we should endorse in‐vitro fertilization (IVF) for different sex‐couples as a way of securing genetic relatedness, as against other ways of helping them have children. So long as this more ambitious critique remains undone, one has to wonder about the merits of skepticism about biogenetics when it is selectively deployed against same‐sex couples. To show as much, I will examine representative views that call into question the value of synthetic gametes and shared maternity for same‐sex couples, before sketching some larger implications of the critique of biologism for social practices that embody and sustain that ideology.

## 2 SYNTHETIC GAMETES

Heidi Mertes decouples genetic relationships between parents and children by saying: “a genetic link is neither a sufficient nor a necessary condition for a valuable parent‐child relationship.”4Mertes, H. (2014). Gamete derivation from stem cells: revisiting the concept of genetic parenthood. *Journal of Medical Ethics,* *40*(11), 44–747. She does say, however, that

I do not wish to deny that there is an added value in parents and children sharing genes. The fact that children resemble their parents creates a bond, the fact that children are made out of their parents’ ‘flesh and blood’ provides a very strong connection, and the idea that a couple’s genomes are mixed together into one new individual is a romantic thought.

But this only to say that “a genetic connection may put you one step ahead, but a good relationship is largely dependent on parenting, not just parenthood.”

Mertes says that these considerations about the moral irrelevance of genetic relatedness may not suffice as reasons sufficient to bar the research and development of synthetic gametes outright but that they nonetheless deserve some attention, an attention that she does not specify. Yet she says forcefully that “it is *important not to reinforce the dogma* that genetic parenthood is ‘the best kind of parenthood’ and to realize that sometimes a gratifying feeling of kinship can be created in a safer and *more acceptable* way than by ‘full’ genetic parenthood” (emphases added). For example, she suggests that partial genetic relatedness to children should be ‘*desirable enough*,’ (emphasis added), as it is to some women with no natural ova who take ova from their sisters in order to conceive. It is to these options that she would direct infertile couples, if not to childlessness altogether.

Mertes does not direct this interpretation of parent‐child relationships at people who are able to conceive and gestate children without any kind of clinical assistance, namely the vast majority of people who aspire to and act to secure genetic relations to their children. Instead, she deploys the interpretation against people who are not able to have genetically related children because of one kind of infertility or another. Mertes says: “In principle, the *optimal scenario* would be that people who are infertile can be convinced that they overestimate the importance of genetic parenthood and that there are other equally valuable means of constructing a family—or building a meaningful life without children—thus *changing their desires*” (emphasis added).

Mertes argues in particular that holding out the prospect of synthetic gametes for same‐sex couples looking to be related to their children in a complementary 50–50% genetic way will only reinforce and sustain “the idea that genetic parenthood *is something that we ought to be pursuing*” (emphasis added). Mertes says that if synthetic gametes were available, lesbian couples “who are now satisfied with donor sperm options might reassess that interest and see it as inferior to “their ‘own’ lab‐created gametes.” Mertes also says “This is not only *a wrong message to send* but also creates a situation in which this new innovation not only meets demand but creates demand” (emphasis added).

Mertes accepts the moral legitimacy of assisted reproduction in principle, as well as its clinical and research subsidy by the state, as a matter of principle. Even so, her account implicitly raises significant barriers to priority in funding—at least as far as the public fisc is involved—as she treats the interest in a broad swath of fertility practices and research as being tainted with biologism. Mertes says that even if one is persuaded that the alleviation of suffering is important (whether for individuals, different‐sex couples, or same‐sex couples), and “Even if one accepts the moral duty of helping infertile people based on this argument, it does not logically follow that all possible means of assisted reproduction should be offered.” She says that when it comes to funding clinical or research efforts, society is entitled to balance those efforts relative to “other needs in society that may have a more convincing claim on the healthcare budget.” Mertes does not specify what criterion distinguishes other claimants as having a “more convincing claim on the healthcare budget,” but presumably it would be those claimants whose purposes were not based on the presupposition that parents should be genetically related to their children as far as possible. There are, of course, various criteria for prioritizing one claim over another relative to state funds, but I will mention just one approach: prioritize funds for diseases and disorders for which there are no good treatments at present over expenditures for diseases and disorders for which there are effective treatments.5Fleck, L. (1994). Just genetics: A problem agenda. In M. Lappé, M. (Ed.), *Justice and the human genome project *(pp. 133–152). Berkeley, CA: University of California Press. On such a view, as there are other ways to have children, one might be hard pressed to make the case that the state ought to subsidize ARTs and/or research that works to ensure parents have genetically related children as far as possible. If one interprets the suffering associated with childlessness as something that belongs within the orbit of health care (as against just bad luck), one can still ask whether adoption might be ‘treatment’ enough, as against fertility treatments properly speaking, let alone fertility treatments subsidized by the state, which subsidy comes at the cost of help for other conditions. In any case, Mertes has raised a very high bar relative to the state subsidy of ARTs by treating infertility as a condition that has no privileged claim on public funds and by suggesting that society has a responsibility to avoid the worst effects of biologism.

For the sake of fleshing out the implications of Mertes’s argument, suppose that zero‐sum calculations were not an issue in public decision‐making. Suppose also that enough government money was available for any and all ARTs and fertility research that people might be interested in. Such financial largesse would, however, be no answer to Mertes’s claim that it is *important not to reinforce* the objectionable dogma of biologism. But Mertes seems not to object in principle to public support of IVF, only to the belief that it has a privileged status, as against being only one claimant among others on the public fisc.6While Mertes makes no mention of this, I will also note that her argument implicitly calls into question the use of private funds that advance biologism, no less than the use of state funds that way, if only because private funding of synthetic gametes would also be complicit in a message *wrong to send. *It may be that there are certain reasons to grant privately funded clinicians and researchers greater liberty than publicly funded clinicians and researchers, but it is unclear that private ventures should be understood as being immune from moral criticism about biologism by reason of the different sources of their funds. If the objection to the dogma of biologism is to have any teeth, it should be able to sustain decisions to withhold financial support for clinical treatments and/or research having genetic relatedness as their goal.

The significance of the anti‐biologism view comes into sharper focus as we consider the matter of synthetic gametes. We cannot now know whether synthetic gametes will ever become a routine clinical option, but we can know that if synthetic gametes go unresearched men would never be able to rely on synthetic sperm (or even ova) and women would never be able to rely on synthetic ova (or even sperm) to have children with those gametes. Mertes is unperturbed about such effects as she sees genetic relationships with children as overvalued and maybe even wrongly valued, but she limits the application of this view to same‐sex couples and not to different‐sex couples more generally. In a separate analysis of which program of synthetic gamete research might best serve the needs of same‐sex couples, Mertes seems to walk away from her own self‐described *optimal scenario* in which same‐sex couples would rein in their expectations of genetic relatedness to children if not the very desire itself for children. Mertes even draws back from her view that endorsing “lab‐created gametes” is “a wrong message to send.” Gone too are concerns that the expectation of synthetic gametes may create demand for novelty in fertility treatments where none previously existed, not to mention extinguishing the demand for ‘good enough’ and possibly already available treatments.

At the outset of this later analysis, Mertes joins Segers et al. in expressing skepticism that the “enterprise of gamete derivation to establish genetic parenthood reinforces the importance accorded to genetic relatedness, thus strengthening the very ‘problem’ that it is meant to solve.”7Segers, S., Mertes, H., Pennings, G., de Wert, G., & Dondorp, W. (2017). Using stem cell derived gametes for same‐sex reproduction: An alternative scenario. *Journal of Medical Ethics,* *43*(10), 688–691. Yet as permission to forge ahead with further discussion of gamete derivation, Segers et al. acknowledge that their skepticism about genetic relatedness will make no headway against people’s actual interests in the kind of children they want. They say that “the preference for a genetically related child seems too entrenched as a matter of both evolutionary biology and culture” to expect that it can simply be set aside as a result of “rational debate”.

After making this concession to people’s deeply entrenched and purportedly irrational preferences, Segers et al. describe possible methods of gamete derivation and argue that one kind is nearer at hand in terms of research development than another for same‐sex couples interested in genetically related children. The nearer prospect would allow a same‐sex couple to share their genes in a child with a 50% genetic contribution from one parent and 25% genetic contribution from the other parent, as against the remoter prospect of sharing parental genes in a child in a 50–50% way. The more likely technique for gamete synthesis would not reflect parity with the standard genetic complement of children of different‐sex couples, but Segers et al. characterize the expectation of 50–50% genetic parentage as very unlikely for same‐sex couples, given current biogenetic limitations in gamete synthesis. Segers et al. therefore endorse the scientifically nearer‐at‐hand prospect by which same‐sex couples might share 50–25% genetic relatedness to children, as against a more remote possibility of the 50–50% genetic relatedness that is available in the children of different‐sex couples.

Taking these accounts together, Mertes both denies that genetic relatedness matters in any morally significant sense but also acknowledges that its perceived value sometimes overrides that moral devaluation, at least in a way that opens the door to some version of synthetic gametes for same‐sex couples. Segers et al. might be right about which option of gamete derivation is most promising, but it is unclear why the expectations of a 50–50% genetic relatedness in children should exceed the moral ambitions and psychological interests of same‐sex couples, as the interest in biogenetic relationships with children is otherwise treated as a bedrock trait of different‐sex couples. As a matter of psycho‐moral expectations, same‐sex couples may prove just as intractable to “rational debate” about the lack of value in biologism as their different‐sex counterparts, and no reason offered by Segers et al. shows why that aspirational goal should be set aside, especially as long as others are given free rein to indulge that ambition with the combined force of *both* evolution *and* culture summoned in their defense.

## 3 SHARED MATERNITY

Guido Pennings has offered a general defense of shared maternity as “acceptable,”8Pennings, G. (2016). Having a child together in lesbian families: Combining gestation and genetics. *Journal of Medical Ethics,* *42*(4), 253–255. meaning by shared maternity two women attempting to have a child together, one of whom offers ova or fertilization while the other gestates the child. For Pennings, the moral acceptability of the practice turns on its analogies with other kinds of morally acceptable assisted reproduction and its perceived value to the women involved. Surprisingly, Pennings says that the acceptability of the practice “does not mean, however, that one should accept this as normal practice.” This judgment seems to be based on the risks of ovulation induction (for one woman) together with the risks of embryo transfer (for the other woman). The overall total risks for a lesbian couple wanting a child could, however, be reduced by relying on intrauterine insemination for one woman, and forgoing ovum donation by the other, although that approach precludes shared maternity.

I do not see in this risk/benefit analysis, however, anything unparalleled in kind in the risk/benefit analyses required in other decisions about ARTs, and maybe not anything different in degree either. In order to increase the odds of securing a single pregnancy, some women, for example, choose to implant multiple embryos, no matter the foreseeable risks of multiple fetuses.9Tremellon, K., Wilkinson, D., & Savulescu, J. (2015). Is mandating elective single embryo transfer ethically justifiable in young women? *Reproductive Biomedicine and Society Online* *1*(2), 81–87. When attempting to have children, many couples grapple with questions of gestating at an advanced maternal age.10Lebovitz, O., Haas, J., James, K.E., Seidman, D.S., Orvieto, R., Hourvitz, A. (2018). The expected cumulative incidence of live births for patients starting IVF at age 41 years or older. *Reproductive Biomedicine Online, 37*(5), 533–541. To mention another example, others wrestle with the question of how many attempts at assisted pregnancy are too many, when calculating total exposure to risk. Against this kind of background, the degree of risks involved in shared maternity seems entirely within the spectrum of ‘normal’ calculations faced by couples seeking to have children.

In his analysis of shared maternity, Ezio Di Nucci also takes as axiomatic the view that parental relationships with children ought to be unstructured by biological relationships, morally speaking; he morally denatures biogenetic relationships with children, saying there is “no intrinsic value in parents’ biological ties to their children.”11Di Nucci, E. (2016). IVF, same‐sex couples, and the value of biological ties. *Journal of Medical Ethics,* *42*(12), 784–787. He maintains that biological ties to children should structure neither the parents’ relationships with one another (e.g., status differentiations according to the kind of biological tie involved) nor parents’ relationships with the children (e.g., differential rights and responsibilities). Armed with the foregoing account, Di Nucci looks in particular at the practice of taking ova from one woman, fertilizing them in vitro, and transferring the subsequent embryos into the woman’s female partner.12For some early ethical analysis of this now decades‐old practice, see Chan, C. (1993). Coupling and maternity. *Ethics and Behavior,* *3*(2), 212–213. Fox, J. (1993). A physician’s perspective. *Ethics and Behavior,* *3*(2), 213–217. Murphy, T. F. (1993) Lesbian mothers and shared maternity. *Ethics and Behavior, 3*(2), 220–224. No matter the value of this kind of shared maternity for the parties involved, Di Nucci characterizes the interest as nothing more than a “wish” because “there is no deeper meaning.” Di Nucci even rejects interpreting shared maternity as a ground of equality between the two women in relation to the child; any such approach would depend on the assumption that differences in biogenetic relatedness justify status difference between partners. As he sees things, biological relatedness to children should play *no role* in the “distribution of roles, responsibility, and ultimately, power.” Di Nucci does not argue that shared maternity is wrong or that it should be illegal. In fact, he stipulates its moral permissibility and even says that any country that would prohibit the practice is “guilty of both incoherence and discrimination.” Even so, he says he only wants to make a point in principle: “Shared biological maternity does not constitute added value” because “biological ties ought not to be meaningful to the prospective parents.”

But if parents leave behind any expectation that biogenetic relatedness structures moral relations in any way, or should, we may well ask whether the state should financially support ARTs undertaken to secure that relatedness. Di Nucci cedes that his analysis might indicate that the state should not offer support for shared maternity, but he says that he doesn’t want to address this question directly because doing so “would take us too far,” as questions about state subsidy for ARTs are not confined to shared maternity. Despite this demurral, Di Nucci does in fact go on to say that the state might pay for interventions enabling shared maternity under some circumstances. He would accept shared maternity being funded for cases having “medical grounds.” For example, a woman unable to become pregnant herself for medical reasons might donate her ova to her partner. In this case the gestating partner would be functioning as a gestational surrogate for the ova donor, with it being only coincidental—from a moral point of view—that the gestational surrogate is also the woman’s same‐sex partner. This kind of example notwithstanding, Di Nucci says he only wants to make a point in principle: that parental genetic relatedness to children does not matter, morally speaking.

Having opened the door to state funding of at least some instances of shared maternity, Di Nucci further allows that it might be difficult in practice to distinguish qualifying instances from others, in which case the state might act to fund all of them rather than get bogged down in fine‐grained distinctions difficult for clinicians and their patients to accept. But if practical concerns make clear distinctions between acceptable (read: fundable) and unacceptable (read: unfundable) subsidies impossible, let me point out that the state’s decision might go the other way; namely, that no one gets funded in regard to fertility interventions if the goal is to secure genetically related children (specifically IVF in the majority of cases and certain instances of intracytoplasmic sperm injection and insemination). Declining to subsidize all these treatments would be just as much a solution to the problem of difficult boundary‐drawing as any other, especially when the interest in having biogenetically related children is simply just wish fulfillment, it is perhaps pernicious relative to the indefensible values it embodies and perpetuates. Declining to subsidize these treatments would also signal in a symbolically important way that genetic relatedness is not an important state goal in helping parents have children.

Let me also note that even if governments or private insurers have some moral obligation to help people have children, nothing about that duty in itself that ostensibly requires them to help people have genetically related children, as against, for example, providing for the adoption of unrelated children or the use of donor gametes and embryos, and this caution would seem to apply even to couples who were infertile for ‘medical reasons’ properly speaking. Di Nucci does allow that this analysis of shared maternity “may have consequences for the funding of traditional IVF too,” but he develops no argument along these lines. This shift away from IVF in general shows all too well the larger implications of anti‐biologism getting lost in the focus on same‐sex couples.

## 4 SOME IMPLICATIONS

At the very least, the anti‐biologism view raises important questions about state funding of ARTs or state requirements that private insurers cover ARTs, not to mention support for research aiming to secure genetic relationships between parent and child. Anti‐biologism calls support for the subsidy of IVF and other infertility treatments into question in a prima facie way, at least in the absence of other arguments that override the moral view that biologism is only a wish, that it is incidental in parent‐child relationships, and its pursuit should not be reinforced, and that it is the cause of harm to some children.

One possible way to align social practices with anti‐biologism views is—at minimum— to withdraw government and insurance support for fertility treatment having as its goal genetically related children; governments might also decline to fund further research aimed at novel ways of ensuring genetically related children. More ambitiously, we might expect government and/or other social institutions not only to defund practices aimed at securing genetically related children but to take active steps to undo the perception that children’s genetic relatedness to parents matters in any important way, for example, by emphasizing adoption as preferable to ARTs. Even if governments and social institutions were to move in either of these directions, I agree with Segers et al. that most people would likely remain strongly interested in genetically related children. I do not think this is because people are not amenable to rational debate, as Segers et al. say, but because the interest in genetic relatedness expresses deeply embedded psycho‐moral values. (Even Plato had to resort to a “noble lie” to justify the idea of raising children without specific knowledge of their parents’ identities and vice versa.13Plato, *Republic*, Book 3, 414e–415c.) Given the value people assign to genetically related children, it is unclear how far governments could succeed in withdrawing support for fertility assistance and research, let alone actively work to undo the influence of biologism, especially as numerous professional associations conceptualize infertility as a medical problem, and not merely a trait without significance for health.14Zegers‐Hochschild, F., Adamson, G. D., de Mouzon, J., Ishihara, O., Mansour, R., Nygren, K., … World Health Organization. (2009). International Committee for Monitoring Assisted Reproductive Technology (ICMART) and the World Health Organization (WHO) revised glossary of ART terminology. *Fertility and Sterility,* 92(5), 1520–1524. In other words, it is hard to see the moral precept of anti‐biologism gaining very much political traction at all.

If—to go the other way—one accepts interest in having genetically related children as a bedrock trait of most human beings (without thereby denigrating other kinds of relationships between parents and children) governments and social institutions seem presumptively obliged to extend the reach of that kind of relationship as far as possible, to the extent public institutions and private insurers have responsibility in this domain. To treat everyone equally under this view, it seems to follow that not only different‐sex couples and same‐sex couples ought to be respected in their interest in genetically related children—and perhaps helped to secure this outcome—but also uncoupled persons as well, perhaps even people interested in multiplex parenting, as well as all men and women incapable of producing gametes for one reason or another.15Cutas has described the value of having more than two parents; along the same lines, one might imagine that three parties want to share genetic relatedness to a child. See Cutas, D. (2011). On triparenting. Is having three committed parents better than having only two? *Journal of Medical Ethics,* *37*(12), 735–738.


If genetic relatedness is taken as a prima facie good, might even more genetic relatedness be better? One might imagine, for example, that a single person might wish to become both the genetic father and the genetic mother of a child, in order to deepen the bond between parent and child.16Cutas, D. (2017). “I am your mother and your father!” In vitro‐derived gametes and the ethics of solo reproduction. *Health Care Analysis,* *25*(4), 354–369. Relevant but separate arguments might justify governments and insurers declining to assume responsibility to help some people have genetically related children, such as potential harm to a child genetically fathered and genetically mothered by the same parent, but on its face, conceding the value of genetic relatedness seems to imply its importance for all, triggering and/or deepening responsibilities to help secure that relatedness. This interpretation seems to prove too much, though, as a guide in helping distinguish the comparative entitlement owed to people as prospective parents; accommodating biologism this way might greatly expand third‐party payers’ responsibilities for the subsidy of clinical treatments and research in fertility medicine, without an obvious upper limit to those responsibilities.

In this discussion of some possible implications, I hope I have shown that critics of biologism have a more ambitious task ahead of them beyond simply deploying concerns about novel fertility interventions to same‐sex couples, which seems an overly restrictive worry, if not also potentially prejudicial.

## 5 CONCLUSIONS

Certain commentators treat as axiomatic the view that parental relationships should not depend—morally speaking—on their genetic relatedness to children. This view has figured to significant effect in discussions about synthetic gametes that could offer same‐sex couples the prospect of children with shared genetics and discussions of two women sharing maternity of a child. In these discussions, commentators do not typically rule out such practices as objectionable in themselves, but they do treat them as morally problematic in their motives and effects. The motives are characterized as being colonized by a morally groundless biologism, and the effects are represented as shoring up that biologism, all in a self‐reinforcing way.

To cut against the alleged ills of biologism, some commentators have made various recommendations: counsel infertile people against seeing biogenetic relationships and/or children as important, authorize governments to withdraw support for fertility practices, and maybe forgo synthetic gamete research altogether. These recommendations have a differential effect on same‐sex couples, while functionally leaving intact the privilege of genetically related children for different‐sex couples.17Orentlicher, D. (2010). Discrimination out of dismissiveness: The example of infertility. *Indiana Law Journal,* *85, *143–186. Even if we grant that prospective parents can be mistaken about the meaning and importance of genetic relatedness to their children, it is not clear why same‐sex couples should forgo biogenetically related children while different‐sex couples remain free to pursue them, sometimes subsidized by the state and private insurers.

What, ultimately, is the moral strength of objections to biologism? If it really is “important not to reinforce the dogma that genetic parenthood is ‘the best kind of parenthood’,” where is the critique of the most numerous and the most socially influential practice in which that dogma holds sway: different‐sex couples conceiving children together with clinical assistance? If biologism is not confronted in its paradigmatic exemplars, how seriously are we to take the claim that it is “important” to undo biologism for one certain subset of people looking to fertility options for help in having children? It may even be wondered whether raising concerns about biologism in regard to same‐sex couples and certain others facing fertility is not that these parties are somehow guiltier of biologism but that gatekeeping functions against biologism are simply possible—i.e., politically palatable—here that are not possible elsewhere.

Raising worries about the biologism of same‐sex couples also seems unlikely to undercut the supposed problem in a gatekeeping meaningful way. Even if *no* same‐sex couples ever turned to synthetic gametes or shared motherhood, the parental quest for genetically related children would continue in the broader population. Biologism is, after all, the praxis of clinically assisted reproduction so far as possible, not to mention the effect of all clinically unassisted conception. Where is the critique of the global practices of fertility medicine as they aid and abet biologism? It may be that some of its critics think that the mountain of biologism is simply too big to move, but I see no reason why criticism of biologism should be focused primarily on same‐sex couples rather than on the parties most in its thrall. So long as we decline to analyze biologism in regard to the people most beholden to it, it becomes proportionately less important to interpret it to the disadvantage of same‐sex couples, or even to interpret it as a meaningful moral criticism at all.

## 6 CONFLICT OF INTEREST

The author declares no conflict of interest.

